# Insulin Receptors and Insulin Action in the Heart: The Effects of Left Ventricular Assist Devices

**DOI:** 10.3390/biom12040578

**Published:** 2022-04-14

**Authors:** Konstantina Pantazi, Eleni Karlafti, Alexandra Bekiaridou, Matthaios Didagelos, Antonios Ziakas, Triantafyllos Didangelos

**Affiliations:** 1 Diabetes Center, 1st Propaedeutic Department of Internal Medicine, Medical School, AHEPA University General Hospital, Aristotle University of Thessaloniki, 54621 Thessaloniki, Greece; konstantinapantazi@yahoo.com (K.P.); linakarlafti@hotmail.com (E.K.); ampekiaridou@gmail.com (A.B.); 2 First Cardiology Department, AHEPA University General Hospital, 54621 Thessaloniki, Greece; manthosdid@yahoo.gr (M.D.); tonyziakas@hotmail.com (A.Z.)

**Keywords:** insulin, insulin receptors, diabetes mellitus, heart, left ventricular assist device

## Abstract

This year, 2022, marks the 100th anniversary of the isolation of human insulin and its administration to patients suffering from diabetes mellitus (DM). Insulin exerts many effects on the human body, including the cardiac tissue. The pathways implicated include the PKB/Akt signaling pathway, the Janus kinase, and the mitogen-activated protein kinase pathway and lead to normal cardiac growth, vascular smooth muscle regulation, and cardiac contractility. This review aims to summarize the existing knowledge and provide new insights on insulin pathways of cardiac tissue, along with the role of left ventricular assist devices on insulin regulation and cardiac function.

## 1. Introduction

Insulin exerts a broad range of effects on the heart, implicating numerous pathways. In other words, the heart is an insulin-dependent organ, meaning that insulin promotes glucose as the primary source of cardiac energy. Insulin signaling affects a variety of myocardium cells, such as cardiomyocytes, fibroblasts, and endothelial cells. As for insulin’s roles, among others, it decreases myocardial O^2^ consumption, improves cardiac efficiency, assists with myocardial relaxation, and promotes increased blood flow to the myocardium [1]. Insulin signaling in the heart is achieved through insulin and IGF-1 receptors, which bind insulin, IGF-1/2, and insulin receptor substrates 1 and 2 (IRS1 and IRS2). These are the main insulin-signaling elements that regulate cellular metabolism [2]. Moreover, the phosphatidylinositide-3-dependent kinase (PI-3K) activity that controls Akt, the PKB/Akt signaling pathway, and the Janus kinase (JAK)2 pathway contribute primarily to the immediate effect of insulin on the heart muscle [2,3].

Insulin secretory dysfunction and insulin resistance can lead to diabetes mellitus (DM), a disease that accounts for increased cardiovascular morbidity, mortality, and healthcare costs. Nowadays, DM has become one of the most common diseases, and its progression can be proven fatal. The prevalence of DM has increased dramatically in all countries, regardless the income levels [4]. Both type 1 and type 2 DM are quite common, with type 2 DM being more frequent, especially in adults (>65 years), while type 1 DM is most common among younger adults (20–44 years) [5]. This year marks the 100th anniversary of the isolation of human insulin and its successful administration to patients suffering from diabetes mellitus (DM). This practice was rather successful, and it sets the mark for the therapeutic use of insulin on patients, impacting the course of the disease.

DM has been associated with several comorbidities, including heart failure (HF), since HF is due to poor glycemic control [6]. Ventricular assist devices (VADs) have been implanted in many patients suffering from advanced HF. VADs are a pivotal solution, either as a destination therapy or as a bridge toward transplantation, and that is because they manage to assist cardiac circulation. Recent research indicates that VADs also help control the glycemic level, leading to lower needs for antidiabetic medication and significant improvements regarding glycated hemoglobin, insulin requirements, and glucose levels [7,8]. This review aims to study insulin receptors further and summarize the effects of insulin on the heart, shedding light on novel insights while also assessing the results of VADs implantation on glycemic metabolism and insulin resistance in patients with advanced HF.

## 2. Diabetic Cardiomyopathy

The development of diabetic cardiomyopathy constitutes the leading cause of mortality in diabetic patients. Diabetic cardiomyopathy is a heart disease independent of hypertension or coronary atherosclerosis. There is a large body of evidence implicating insulin deficiency/resistance in the pathogenesis of these disorders. Other than diabetic cardiomyopathy, cardiovascular autonomic neuropathy is also a risk factor for patients with DM, as it leads to increased mortality and left ventricular diastolic and systolic dysfunction. As a matter of fact, research has proven that DM type 1 patients with diabetic autonomic neuropathy have a reduced left ventricular filling pattern, with a more intense LV working load and systolic function [9], while DM type 2 patients with diabetic autonomic neuropathy appeared to have an increased working LV workload, as well as diastolic dysfunction and an increased A/V index [10].

Diabetic myocardium exhibits characteristic fibrosis, interstitial, as well as perivascular, even when coronary disease and hypertension may be absent. Insulin has effects on the myocardium and the endothelium through multiple important pathways and mechanisms, which are analyzed below. Any occurring malfunctions of these mechanisms or abnormalities of the molecules that partake in them can have detrimental results. It is also important to note that most of the studies mentioned, which are related to the action of insulin on the heart and IRs’ regulation, have been performed on animal models.

## 3. The Pathophysiological Pathways of Insulin Action Implicate Akt-mTOR, eNOS, and grk2

**Figure 1:**
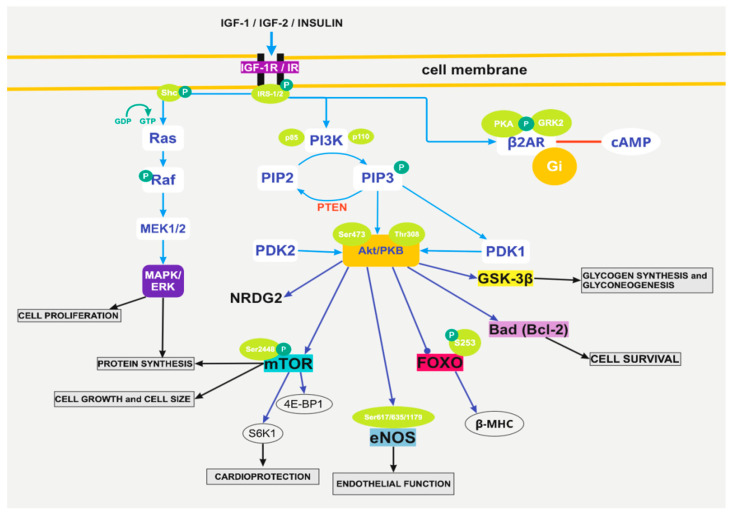
Pathways of insulin signaling in the myocardium. The first step is the activation of the receptors (IGF-1R or IR) by insulin, IGF-1, and IGF-2. All molecules can bind to all receptors, but insulin has a high affinity to the IR, while IGF-1 and IGF-2 have a high affinity to the IGF-1R. After the activation of the receptor, many downstream pathways are subsequently activated, aiming to provide, among others, protein synthesis, cardioprotection, cell growth and size regulation, cell survival, glycogen synthesis, and glyconeogenesis. Abbreviations used: IGF-1: Insulin-like Growth Factor-1, IGF-2: Insulin-like Growth Factor-2, IGF-1R: Insulin-like Growth Factor-1 Receptor, IR: Insulin Receptor, IRS-1/2: Insulin Receptor Substrate 1/2, P: Phosphorylation, Shc: Src homology and collagen adaptor protein, GDP: Guanosine Diphosphate, GTP: Guanosine Triphosphate, Ras: Ras kinase, Raf: Raf kinase, MEK1/2/MAPK: Mitogen Activated Protein Kinase, ERK: Extracellular-signal-regulated Kinase, p85: p85 regulatory subunit, p110: p110 catalytic subunit, PI3K: Phosphoinositide 3-Kinase, PIP2: Phosphatidylinositol 4,5-bisphosphate, PIP3: Phosphatidylinositol (3,4,5)-triphosphate, PTEN: Phosphatase and Tensin Homolog, Ser473: Serine 473, Thr308: Threonine 308, Akt/PKB: serine/threonine kinase/protein kinase B, PDK1: Phosphoinositide-Dependent Kinase 1, PDK2: Phosphoinositide-Dependent Kinase 2, NRDG2: NDRG2 gene, mTOR: mammalian target of rapamycin (protein kinase), Ser2448: Serine 24448, 4E-BP1: 4E Binding Protein 1, S6K1: protein S6 Kinase 1, Ser617/635/1179: Serine 617, 635, 1179, eNOS: endothelial Nitric Oxide Synthase FOXO: Forkhead transcription Factors, S253: phosphorylation site, β-MHC: β-Myosin Heavy Chain, Bad: Bcl-2 agonist, Bcl-2: B-cell lymphoma 2, GSK-3β: Glycogen Synthase Kinase 3β, β2AR: β2-Adrenergic Receptors, PKA: Protein Kinase A, Gi: inhibitory G protein, GRK2: G protein-coupled Receptor Kinase 2, cAMP: cyclic Adenosine Monophosphate.

Cardiomyocyte death results from numerous cardiac injuries and has a determining role in the development of heart diseases. Therefore, cardioprotection is rather essential and is linked to many molecular and biochemical changes [11]. Specifically, when it comes to insulin and IGF-1, they have anti-apoptotic effects on the heart via mechanisms, some of which are dependent on glucose. These anti-apoptotic signals consist of multiple pathways, with one of the most important being the activation of PI3K/Akt. This pathway then triggers various downstream proteins, such as the mammalian target of rapamycin (mTOR), endothelial nitric oxide synthase (eNOS), glycogen synthase kinase (GSK)-3β, forkhead transcription factors (FOXOs), and certain Bcl-2 family members (Figure 1).

### 3.1. The Activation of PI3K/Akt

There are many classes of PI3Ks, each of which has a different structure and mode of activation [11]. More specifically, class I PI3K is divided into I_A_ and I_B_ because of the different binding subunit p110. Then, class I_A_ PI3K catalytic subunits include p110α, p110β, p110δ, and the regulatory subunit is mainly p85α, and class I_B_ PI3K consists of catalytic subunit p110γ, which is regulated with regulator protein p101. In addition, the class I_B_ PI3K can be activated by the G-protein-coupled receptor (GPCR)-β, γ, subunits on p110 activation. As for Akt, mammalian genomes contain three Akt genes that encode the following isoforms: Akt1, Akt2, and Akt3.

Meanwhile, Akt/PKB is the main regulator of the signaling pathways, and it determines numerous cellular functions [11]. Akt kinase has a significant action in the cardiovascular system, such as proliferation and cell growth via mTORC1, promotes cell survival via caspase-9, YAP, Bcl-2, and Bcl-x activities, and angiogenesis, vasorelaxation, and cell metabolism via VEGF secretion and mediates eNOS phosphorylation (Figure 1).

Consequently, the alterations of Akt signaling play an important role in many cardiovascular pathological processes such as atherosclerosis, cardiac hypertrophy, and vascular remodeling.

The activation of the PI3K/Akt is a very important step for insulin to have a cardioprotective effect. This mechanism is a response triggered by a wide range of stimuli, such as insulin, insulin-like growth factor-1 (IGF-1), AT II, and reactive oxygen species (ROS) [11]. It is important to mention that Akt activation by insulin is mediated via tyrosine kinase activity of the insulin receptor (IR), IRS-1, and IRS-2.

At first, either insulin attaches to the insulin receptor or IGF-1/2 attaches to the IGF-1 receptor to activate the receptor (Figure 1). As a result of the activation, PI3K binds to the receptor as well through its p85α subunit and then activates I_A_ PI3K. The latter can also be activated by insulin through IRS-1 and by other tyrosine kinase receptors or cytokine receptors. As for the I_B_ PI3K, it is activated by GPCRs, such as adenosine and opioid receptors. The activation of PI3K subsequently activates the synthesis of PIP3 and phosphorylates phosphatidylinositol 4,5-bisphospate (PIP2), which can regenerate PIP3. After that, PIP3 can recruit phosphoinositide-dependent kinase (PDK)-1 and Akt, which leads to the phosphorylation of Akt and thus its activation [11]. It is also important to mention that the phosphorylation of Thr308 and Ser473 is needed for Akt to be fully activated (Figure 1). Moreover, Akt may also have the ability of autophosphorylation and to be activated independently of PI3K [11].

There are also mechanisms that negatively regulate Akt and PI3K, such as the regulation from phosphatase and tensin homolog deleted on chromosome 10 (PTEN), which converts PIP3 to PIP2 and blocks the activation of the pathway (Figure 1). For this reason, the reduction in PTEN can lead to activation of the pathway and therefore enhanced cardioprotection. Insulin or IGF-1/2 activation of the PI3K/Akt cascade is protective of the heart by inhibiting apoptosis and oxidative stress. While PI3-K and the atypical protein kinase C family (ζ and λ) are responsible for insulin-induced Glut4 translocation in 3T3-L1 adipocytes and L6 myocytes, Akt1 and Akt2 activity may be responsible for activating glycogen synthase [12].

Akt protein is a serine/threonine kinase and a downstream effector of PI3-K. Akt plays a central role in the metabolic actions of insulin, including glucose transport, and the synthesis of glycogen [12]. Maintaining precise physiological levels of Akt/PKB may be critical to avoiding insulin resistance. This is evidenced by studies linking impaired Akt expression and activity with type 2 diabetes [12].

The regulation of cell size by Akt is thought to be mediated by its phosphorylation and by the subsequent downstream phosphorylation of mTOR on the serine 2448 [13]. In general, the PI3K/Akt pathway manages to protect the heart via several impending signaling molecules, such as eNOS, FOXO, Bad, GSK-3β, mTOR, N-myc downstream regulated gene 2 (NDRG2) [11] (Figure 1). For example, the regulation of cell size by Akt is thought to be mediated by its phosphorylation and by the subsequent downstream phosphorylation of mTOR on the serine 2448 [13]. mTOR contributes to the deterrence of cardiac dysfunction in pathological hypertrophy [11]. More specifically, mTOR stimulates cell growth and metabolism and inhibits excessive hypertrophy, thus conferring cardioprotection and cardiomyocyte survival. mTOR’s down activators are two important regulators: p70 S6 kinase 1 (S6K1) and the 4E binding protein 1 (4E-BP1). Out of the two, S6K1 is rather important since the PI3K/AKT/mTOR/S6K1 pathway has an essential share in cardioprotection induced by insulin [11] (Figure 1). As a matter of fact, the excessive activation of mTOR/S6K1 can induce cardiac insulin resistance, while the same pathway can also provide cardioprotection via increased angiotensin II (Ang II) type 2 receptor (AT2R) upregulation and adaptive hypertrophy [14].

Other than mTOR, FOXOs’ phosphorylation has a cardioprotective role by restricting their transcriptional activities [11]. Moreover, Bad (one of the Bcl-2 family proteins), if maintained phosphorylated, manages to suppress apoptosis and promote cell survival [11]. GSK-3β is also essential since it is a serine/kinase that plays an important role in the regulation of glycogen synthesis and gluconeogenesis [11] (Figure 1).

### 3.2. The Pathway of Foxo1

Insulin binds to an IR, while IGF-1/2 binds to an IGF-1R, either of which can phosphorylate, among others, IRS1, which induces downstream cascades, such as PI3Ks and MAPKs [15]. The PI3K/Akt pathway phosphorylates multiple pathways, with one of them being the forkhead transcription factor Foxo1. Specifically, Akt phosphorylates Foxo1 at S^253^ and constrains Foxo1’s activity, which normally is to regulate physiological functions such as myocardial growth [15] (Figure 1).

IRS1 and IRS2 are vital components of insulin signaling, and the loss of IRS1 and IRS2 mediates insulin resistance [15]. In the heart, this results in metabolic dysregulation and heart failure [16], which is interlinked with Akt inactivation and activation of Foxo1. It is important to mention that a decrease in IRS1 and IRS2 with an accompanying activation in Foxo1 is also present in the heart of animals with DM type 2 or insulin resistance [17]. In other words, the parallel inactivation of Akt, activation of Foxo1, and loss of IRS1 and IRS2 are the basis of insulin-resistant cardiomyopathy [15]. Foxo1 also triggers β-MHC gene expression in cardiomyocytes. β-MHC is a target of insulin signaling, and it manages to quell its expression via PI3K activation [15] (Figure 1).

### 3.3. Insulin Growth Factor 1

Insulin-like growth factor 1 (IGF-1) is a single-chain polypeptide that is highly homologous to proinsulin. It is produced in numerous cell types and has both an autocrine and paracrine action. Its activity is arbitrated through binding to the IGF-1 receptor, which strongly binds IGF-1 and IGF-2 but has a low affinity to insulin [18]. In order for the IGF-1 receptor to be activated, the phosphorylation of IRS is mandatory [19]. Tyrosine-phosphorylated IRS-1 and IRS-2 interact with proteins that contain SH2 domains, guiding to numerous cascade pathways. It is also important to mention the IGF-1 binding proteins (IGFBPs), which bind to IGF-1 and manage IGF-1’s binding to the receptor and thus its activity [20].

The IGF-1 signaling cascades are enabled by interactions that lead to the activation of Ras, serine/threonine kinase Raf, and MAP kinase (Figure 1). The latter enables multiple transcription factors and therefore is involved in the IGF-1 stimulation of DNA synthesis and mitogenesis. PI3K is also vital for the metabolic growth and functional effects that insulin and IGF-1 cause, which, among others, include protein and glucose synthesis, apoptosis, and IGF-1-mediated cardiomyocyte contractility [18].

IGF-1 plays an essential role in both cardiac growth and function. When it comes to cardiac growth, IGF-1 boosts cardiac DNA and protein synthesis in isolated cardiomyocytes [21]. Specifically, IGF-1 regulates the cell cycle since, without IGF-1, the cell cannot enter the S phase [22]. Moreover, IGF-1 is associated with hypertrophy and promotes growth through signaling pathways, which include tyrosine kinase, IRS-1, PI3K, and ERK [19].

IGF-1 is both a protective and a risk factor for the heart, and even though IGF-1’s role in cardiac function still remains unclear [18], it has quite the potential. For example, IGF-1 is very likely to be used as a treatment for cardiac disorders (e.g., heart failure) since it can upgrade myocardial function both in cardiac patients and healthy adults [18]. Other than that, IGF-1 may improve cardiac contractility by increasing the synthesis of contractile proteins, while at the same time, multiple IGF-1-mediated signaling pathways, such as tyrosine kinase, tyrosine kinase phosphatase, PI3K, and protein kinase C, have also been involved [23]. The activation of the pathways mentioned can lead to an increase in intracellular Ca^2+^, which is followed by an acute positive myocardial response [18], and to the involvement of ion channels, such as T-type calcium channels [24] and cardiac K^+^ channels [25].

IGF-1 can also affect the heart by preventing apoptosis [26]. This mechanism is based on signaling pathways such as tyrosine kinase, MAP kinase, and PI3K, as well as the increased expression of a member of the anti-apoptosis family of Bcl-2 proteins. IGF-1 may act as a survival factor via stimulation of the Bcl-2 family of proteins [18]. It is also important to refer to the impact of IGF-1 deficiency on the heart. Growth hormone (GH) is essential for the heart to maintain its structure and function. In humans, deficiency of IGF-1/GH with obtained GH resistance is highly related to an increased risk of cardiovascular disease [18].

In addition to everything mentioned, IGF-1 betters blood glucose control and improves insulin sensitivity. As a matter of fact, diabetes is characterized by hepatic GH resistance and high levels of IGFBPs, which result in decreased levels of total and free IGF-1 levels. In diabetes, there is also a resistance to IGF-1-mediated myocardial responses, which can be because of increased NOS activity or because of altered protein tyrosine phosphatase activity. Untypical IGF-1 activity is very likely to be associated with diabetes-related vascular disorders and may even lead to cardiac dysfunction, but it is still obscure.

IGF-1 levels and blood pressure are positively interrelated [27]. In hypertensive/hypertrophic states, there is a broad expression of IGF-1 and IGF-1 receptors. This hints that IGF-1 may be involved in pathways that lead to cardiac mechanical dysfunction. Even though insulin/IGF-1 may not play a role in hypertension, insulin/IGF-1 resistance seems to cause hypertension, especially when IGF-1 is associated with excessive NO levels in both vessels and the heart [28].

### 3.4. The Pathway of eNOS

eNOS, which stands for endothelial nitric oxide synthase, is usually triggered by pathophysiological stimuli, such as vascular endothelial growth factor (VEGF) and stress and is regulated by protein interactions and phosphorylation. eNOS produces nitric oxide (NO) in the endothelium, which is a very important vasoactive compound that also regulates a number of physiological and cellular mechanisms, such as angiogenesis and thrombosis [29]. The regulation of NO’s expression by eNOS is rather important since NO is also highly reactive.

eNOS is a calmodulin-dependent enzyme [30]. Its activation is a result of a rise in intracellular calcium and the subsequent activation of the CaM-binding domain by calmodulin [31]. Its activity is triggered by acetylcholine, bradykinin B2 receptor, thrombin, and ATP, via intracellular elevation of calcium levels [32], and it is also dependent on protein phosphorylation and dephosphorylation. Specifically, the phosphorylation of Ser617, 635, and 1179 leads to activated eNOS, while the phosphorylation of Ser116 and Thr497 results in a decrease in functional eNOS [29]. The serine/threonine kinase Akt phosphorylates Ser1177 and Ser1179 [29].

Insulin exerts a significant cardiovascular protective effect via PI3K/Akt/eNOS-dependent signaling in addition to its metabolic modulation [11] (Figure 1). A dysfunction in the Akt/eNOS signaling pathway can majorly affect endothelial function in diabetes mellitus (DM) type 2. This is a result of a reduction in the Akt/eNOS phosphorylation [29]. Meanwhile, insulin binds to insulin receptors or IGFs bind to IGFRs, and signaling pathways are activated, such as the PI3K/Akt/PKB pathway, which mediates many insulin responses, such as myocardial survival and anti-apoptotic effects in endothelial cells. Therefore, the activation of the PI3K/Akt pathway by NO, derived by eNOS, can lead to enhanced endothelial function [33]. Studies have also shown that eNOS’s activation is stimulated by insulin and is dependent on the phosphorylation of insulin-responsive cells [34].

NO-based therapies have been researched [35], and NO partakes in the diabetic pathology. Data have pointed out that endothelial dysfunction, abnormal eNOS expression, and NO production are characteristics of diabetes and insulin resistance [29].

### 3.5. The Action of G-Protein-Coupled Kinase 2

When insulin activates an IR or IGF-1/2 activates an IGF-1R, protein kinase A (PKA) and G-protein receptor kinase 2 (GRK2) phosphorylation of β2AR are induced (Figure 1). This reaction mediates β2AR’s disassociation from the complex and its binding to the inhibitory G-protein Gi [36]. This last binding manages to alleviate adrenergic-induced cAMP activities in the heart. The phosphorylation of β2AR depends on insulin receptor substrates 1, 2 (IRS1 IRS2). As a result, the IR-β2AR interference results in decreased β-adrenergic-induced contractile function in cardiomyocytes and perfused mouse hearts [36].

Both in diabetes and heart failure, there is an elevation of insulin levels, which constantly stimulates IRs. In DM type 2, the IRs of the heart are still sensitive to and are activated by insulin. These elevated levels of insulin can acutely impair β-adrenergic signaling pathways for contractile function in animal hearts [36].

G-protein-coupled kinase 2 (GRK2) has been proven to be a negative controller of insulin receptor signaling [37]. Even though the molecular basis of GRK2 upregulation’s negative effects is still unclear, it has been proven that its inhibition in animals has led to the prevention of the development of metabolic disorders, modulating energy expenditure, brown fat function, and insulin actions in peripheral tissues [37]. In these animals, the insulin sensitivity was improved, and they displayed enhanced activation of the insulin mediated Akt pathway in muscle, adipose tissue, and liver [37].

Increased cardiac GRK2 plays a pivotal role in cardiovascular diseases such as heart failure and cardiac ischemia, and this is the reason why its inhibition in mice results in cardioprotection [37]. Other than that, increased GRK2 levels are not only a characteristic of lower cardiac functions and poorer prognosis in HF but also a characteristic of situations of systemic insulin resistance. GRK2 is also capable of inhibiting the IR pathway and regulating the adrenergic receptor signaling and thus has the ability to control body weight gain, adiposity, metabolic rate, and multiple downstream targets of the insulin cascade [38].

Decreased GRK2 levels have a cardioprotective role in many animals and maintain metabolic and pro-survival signals downstream of insulin (such as the Akt/p70S6K pathway and glucose transport) in the hearts of 9-month-old GRK2+/− animals, while the activation of ERK is not affected [37]. In adult mice, downregulation of GRK2 ignites physiological heart hypertrophy and activates a cardioprotective gene expression pathway. Moreover, this downregulation also amplifies the insulin triggered PI3K/Akt pathway [37].

The genetic deletion of GRK2 can result in a reverse of diet-induced obesity and insulin resistance [38]. More specifically, tamoxifen-induced GRK2 ablation in mice leads to a reduction in body weight gain despite the ongoing high-fat diet (HFD) feeding, higher insulin sensitivity, regulated glucose intolerance, and the prevention of adiposity and fatty liver [38].

These effects are based on tissue-specific processes such as improved insulin signaling in peripheral tissues, improved lipolysis in white adipose tissue (WAT) and brown adipose tissue (BAT), elevated expression of mRNAs that encode proteins partaking in fatty acid oxidation and thermogenesis in BAT, and lower levels of steatosis and liver inflammation [38].

## 4. Insulin Signaling and Endothelial Function

Other than the effects of insulin on cardiomyocytes, insulin signaling seems to have an important role in endothelial cells as well. As a matter of fact, a dysfunction in the insulin-stimulated endothelial function seems to be associated with diabetic vascular disease [39].

In general, the endothelium contributes majorly to most body structures and functions. For instance, when it comes to endothelial ATP generation in normoglycemic conditions, endothelial cells are mainly glycolytic, and as a result, oxidative phosphorylation contributes only around 15% to endothelial ATP production [40]. Data as such lead to the conclusion that endothelial cell metabolism can be used as a target when it comes to treating diseases [40].

When it comes to DM, it is believed that diabetes associated with insulin resistance leads to inflammatory changes such as the production of cytokines, adhesion molecules, and reactive oxygen species [41]. Insulin’s role in the endothelium assists with the suppression of these changes, either by decreasing glucose levels or stimulating nitric oxide (NO) production and inhibiting anti-inflammatory pathways. Insulin controls NO production by regulating the enzyme NO synthase, which is responsible for the conversion of arginine to NO [41]. It is important to mention that insulin induces vasorelaxation by NO both in rats and in healthy humans [39]. Moreover, insulin decreases inflammation by diminishing plasma concentrations of adhesion molecule production in endothelial cells [41].

**Figure 2:**
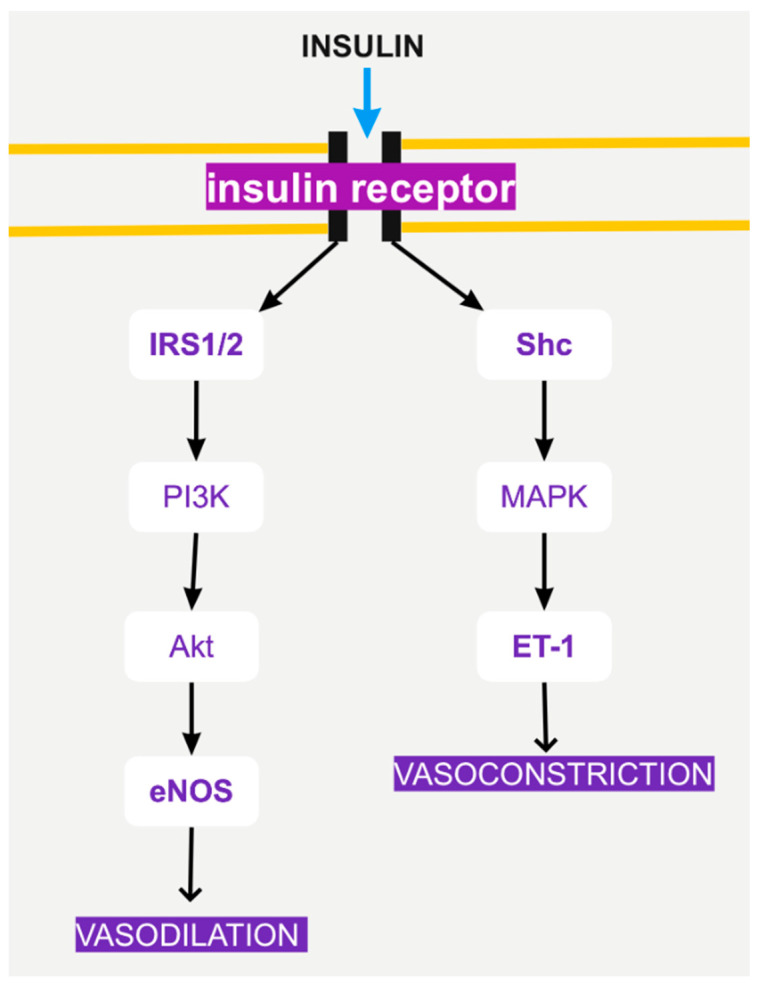
Insulin-signaling pathways in endothelial cells. Insulin can lead to either vasodilation or vasoconstriction, depending on the pathway.

More specifically, insulin induces NO-dependent vasodilator actions and endothelin-1 (ET-1)-dependent vasoconstrictor actions [42] (Figure 2). These actions are regulated by PI3K and MAPK signaling in the vascular endothelium (Figure 2). The balance between the PI3K and MAPK pathways is responsible for regulating the secretion of the ET-1 and determines the vascular response to insulin [42].

It is also interesting to mention that the endothelium is highly sensitive to the toxicity caused by glucose [40]. More specifically, it is believed that hyperglycemia leads to an imbalance of expression of the transporters and that the ejection of glucose from the endothelium is jeopardized, thus resulting in increased glucose concentration in endothelial cells and higher intracellular levels of ROS [40].

## 5. Genetic Loss of Insulin Receptor

Genetic loss of insulin receptors worsens cardiac efficiency in diabetes [43]. Research performed on mice with the genetic absence of insulin receptors in cardiomyocytes (CIRKO) proved that when diabetes was induced, the already existing mitochondrial malfunction and uncoupling in the hearts were associated with a rise in myocardial oxygen consumption (MVO2) and a reduction in cardiac efficiency (CE) [43]. Rather interesting is the fact that mitochondrial dysfunction can occur independently due to alteration in circulating levels of glucose, but the levels of CE are more likely to change when accompanied by mitochondrial dysfunctions. In other words, an antecedent mitochondrial dysfunction predisposes to the reduction in CE in diabetes [43].

CIRCO mice are characterized by mitochondrial dysfunction [43]. In CIRCO mice, the changes in mitochondrial function and CE occur as they become older in age, meaning that younger mice have increased MVO2 and fatty acid oxidation (FAO). Moreover, hyperglycemia in CIRKO mice causes a dramatic increase in MVO2 and a reduction in CE [43].

Moreover, the genetic deletion of IGF-1R worsens cardiac efficiency as well. Research performed on cardiomyocyte-specific IGF-1R knockout (CIGF1RKO) mice has proven that irreversible deletion of IGF-1R in cardiomyocytes prevents structural deterioration since the continuous expression of IGF-1 finally leads to pathological hypertrophy [44]. It has also been shown that genetic deletion of IGF-1R also leads to a reduction in exercise-induced cardiac hypertrophy [44]. Other than that, researchers pointed out that even though in CIGF1RKO mice fibrosis was decreased, the systolic rate of the heart remained normal, probably due to a boosted ability to adjust to hemodynamic stress [44]. Some other interesting findings are the fact that proinflammatory cytokines in the heart are partially stimulated by the IGF-1R and the fact that even though phosphorylation of Akt was increased in old hearts of normal mice, it was not in old CIGF1RKO mice hearts. This could mean that the IGF-1R-Akt cascade leads to cardiac aging [44].

## 6. The Role of Sacro/Endoplasmic Reticulum Ca^2+^-ATPase (SERCA)

DM is often accompanied by heart disease, such as diabetic cardiomyopathy. This disease is characterized by alteration in ventricular myocytes and mechanical dysfunctions. Specifically, it involves irregular myocyte excitation-contraction (E-C) coupling, decreased expression and function of sacro/endoplasmic reticulum Ca^2+^-ATPase (SERCA), and Na^+^/Ca^2+^ exchange (NCX) [45].

When exploring cellular mechanisms in cardiomyocyte dysfunction in insulin-resistant rats, the results show that slowed cytosolic Ca^2+^ removal and myocyte relaxation in cells from SU-fed rats contain slowed SR Ca^2+^ uptake and no change in SERCA2a protein content [45]. It is also proven that NXC and PLB content, as well as the extent of phosphorylation, are actually normal in dysfunctional cardiomyocytes [45]. Malfunctioning cardiomyocytes are also characterized by changes in the regulation of intracellular Ca^2+^, which can occur not only in diabetes but also in mild metabolic disorders such as mild insulin resistance [45]. At this stage, the cardiomyocyte malfunction is reversible. In other words, cardiomyocyte dysfunction in diet-induced insulin-resistant rats usually contains depressed SECRA activity, which leads to impaired relaxation, but no impact on NCX and no changes in SECRA2a, NCX, or PLB protein content [45].

It is important to mention that contractile dysfunction and depressed SECRA activity commonly occur in DM type 1 [46] but not always in DM type 2. SECRA activity is suppressed, with a simultaneous change in the mRNA that codes the protein, in ventricular tissue isolated from glucose-intolerant rats, in genetically obese rats [47], and in Otsuka Long–Evans Tokushima fatty rats [48,49], but not in JCR:LA-cp rats [49]. Data show that there can be changes in SECRA function without an accompanying change in SECRA protein levels in myocytes maintained in a high-glucose medium [45].

## 7. Chronic Hyperinsulinemia and Angiotensin II (Ang II)

Angiotensin II (Ang II) is the active part of the renin-angiotensin system (RAS) and is essential in cardiomyocyte hypertrophy and cardiac fibrosis [50]. Ang II binds to two angiotensin II receptors (AT-R): AT1-R and AT2-R. The first one is in charge of increasing norepinephrine release and thus the rate and force of cardiac contraction and myocardial cell growth. AT1-R’s antagonists manage to decrease cardiac hypertrophy in animals and hypertensive patients. Insulin triggers the RAS pathway and leads to overexpression of AT1-R and subsequently enhances the efficacy of Ang II in animal models [50].

Hyperinsulinemia is characterized by abnormally elevated levels of insulin in the body. Hyperinsulinemia increases AT1-R and AT2-R levels. Moreover, bearing in mind that insulin also stimulates growth and triggers many signaling cascades, hyperinsulinemia boosts the serine phosphorylation of IRS-1 (Ser375), MEK1/2, ERK1/2, S6K1, and PI3K p110a, decreases tyrosine phosphorylation IRS-1, and the p85 subunit of PI3K is not changed. Other than that, it causes myocyte hypertrophy and increased interstitial fibrosis [50].

However, insulin administration at doses that maintain the plasma glucose within the normal range significantly reduces many factors predisposing to atherothrombosis, such as intracellular adhesion molecule-1, chemo-attractive monocyte protein-1, metalloproteases-2 and -9, and PAI-16.

## 8. Insulin-Mediated Glucose Transport via Glut

Insulin and glucagon are two hormones responsible for the maintenance of plasma glucose levels throughout the day [51]. Specifically, insulin causes GLUT4 translocation and increases the activation of GLUT4 transporters in the heart. Insulin also stimulates the translocation of GLUT1 in cardiomyocytes [52].

### 8.1. GLUT-1

GLUT1 is mainly dominant in the embryonic heart, but the ratio of GLUT1/GLUT4 becomes level shortly after birth [52]. In the heart, the main regulators of GLUT1 are the SP1 and SP3 transcription factors. To be more exact, SP1 is a positive regulator, while SP3 negatively affects GLUT1’s expression. It is important to mention that the decrease in glucose and insulin levels and the simultaneous increase in fatty acid levels that is observed during fasting are associated with a decline in GLUT1 expression. At the same time, the GLUT4 expression levels remain the same, which indicates that GLUT4 is the heart’s main regulator of insulin-mediated glucose uptake. However, there have been circumstances when GLUT1 levels increase, as, for example, in rats with chronic left ventricular hypertrophy (LVH) and during inflammatory myocarditis [52].

### 8.2. GLUT-4

As mentioned above, GLUT4 plays an essential role in insulin-mediated glucose uptake. More specifically, insulin manages to convert glucose uptake into fat and muscle cells through the transportation of glucose transporter type 4 (GLUT4) [51]. When insulin is absent, GLUT4 is misdistributed, and it fails to transport to the plasma membrane, which leads to early signs of insulin resistance and DM type 2 [51].

Insulin-regulated glucose transport is mediated by the PI3K pathway and an APS (adaptor protein with pleckstrin homology (PH) and Src homology 2 (SH2) domains) signaling cascade [51]. These two pathways regulate GLUT4’s transportation via multiple cascades, which contain lipids, protein and lipid kinases, small GTPases, and adaptor proteins [51]. The PI3K pathway consists of insulin’s binding to an IR, the recruitment of an IRS, the subsequent activation of PI3K, and the synthesis of PtdIns(3,4,5)P_3_. Following up, PDK1 and mTORC2 activate AKT, which plays a pivotal role in connecting insulin signaling with regulators of GLUT4 transport. The targets of AKT are some small GTPases and some SNARE regulatory proteins, which regulate GLUT4’s trafficking [51].

Research has proven that GLUT4 translocation in skeletal muscle and the heart is stimulated by bradykinin. Bradykinin enhances the phosphorylation of IRs, IRS-1s, and PI3Ks in the skeletal muscle of aged rats. All these molecules are necessary for insulin-stimulated GLUT4 translocation and glucose transport. This also shows that the amount of GLUT4 is related to blood pressure levels [53].

Other than the PI3K pathway, insulin also regulates the adaptor protein APS pathway. This protein binds to the IR, and following its phosphorylation, a cascade of molecules occurs, which finally leads to GLUT4 exocytosis and transportation [51].

It is also interesting to mention that myocardial ischemia tends to induce the translocation of GLUT4 and GLUT1 to the sarcolemma and is associated with an increase in glucose uptake. In this case, an activated AMP kinase (AMPK) plays a vital role in the response since, in studies in mice, it was proven to block hypoxia, assist in GLUT translocation, and control glucose uptake.

## 9. Adaptations in Cardiomyocytes under Conditions of Insulin Resistance

Insulin resistance implies that insulin levels are lower than expected by the body, therefore leading to various effects on different tissues depending on the metabolic demands. For example, insulin resistance causes endothelial dysfunction by limiting the amount of NO that is being produced. Insulin resistance in cardiomyocytes affects the PI3K pathway, while the MAPK cascade remains intact [54]. When insulin resistance affects the binding of IRS-1 to the receptor, it leads to reduced glucose trafficking and phosphorylation and decreased NO endothelial function. This also leads to hyperinsulinemia, which overstimulates the MAPK pathway and leads to inflammation. In other words, when insulin resistance affects the PI3K pathway, the MAPK pathway remains intact and activates numerous inflammatory cascades, such as inhibitor kB (IkB)/nuclear factor kB (NFkB) and c-Jun N-terminal kinase (JNK), which also cause insulin resistance [55].

Insulin signaling controls glucose and lipids metabolism in the heart. When insulin resistance occurs, the result is high lipid oxidation and low glucose oxidation because of lipotoxicity caused by the elevated FFA uptake [54]. Moreover, the activation of RAAS can lead to mitochondrial dysfunction and oxidative stress, which subsequently activates the mTOR/S6K1 signal and perpetuates insulin resistance [54].

## 10. Ventricular Assist Device Implantation Reverses Insulin Resistance

### 10.1. HF and DM Association

Heart failure (HF) is strongly associated with DM, and studies have proven that there is a higher risk of HF in diabetic men and women [7]. Poor glycemic control, autonomic nervous system dysfunction, hypertension, and dyslipidemia are major risk factors for HF in patients with DM. The patients treated with the intensified, multifactorial intervention of all the previously mentioned factors for the initial 7.8 years exhibited a markedly (about 70%) lower risk of hospitalization for heart failure [56] and a substantial increase in lifespan (by a median of 7.9 years), matched by time free from incident cardiovascular disease [57]. Hospitalization for patients with HF tends to be very difficult since the circumstances are very demanding and challenging [57]. As a matter of fact, advanced HF, due to poor perfusion, causes neurohormonal imbalances (increase in cortisol and catecholamine levels [7]) and inadequate hemodynamics, which increase insulin resistance and deteriorate glycemic control [58].

HF and PO (pressure overload) cardiac hypertrophy are associated with DM type 2 and the subsequent insulin resistance and hyperinsulinemia. Research on mice and humans has proven that DM type 2 can be associated with pathological left ventricular remodeling and, more specifically, that the remodeling is due to hyperactivation of IRS1 and Akt1 signaling (and is irrelevant to the IRS2 and Akt2 signaling). In other words, the IRS-1 stimulated cascade plays a vital role in HF and its association with insulin signaling, both in mice and in humans [59].

### 10.2. Pharmacological Treatment of DM Type 2 in Patients with Cardiovascular Disease

Nowadays, the optimal treatment strategy for patients with DM type 2 and HF remains controversial [60]. However, it is widely believed that metabolic interventions that can enhance glucose metabolism, such as SGLT2 medications, are the most beneficial [60,61]. In other words, glucose-lowering therapies, which contain the elimination of glucose through the kidney, seem to be the most effective solution [62]. Moreover, evidence shows the benefits of the use of metformin, which lowers endogenous glucose production and has been proven to be safe and effective [60]. Other therapies that target DM type 2 in patients with HF include RAS blockade [61], which can control blood pressure, and statin treatment [61], which is responsible for lipid control. These therapies decrease the morbidity of cardiovascular diseases [61]. It is also important to mention that when it comes to insulin, its inhibition in patients with DM and HF should be closely monitored [63], as insulin leads to sodium retention, which can worsen cardiovascular conditions.

### 10.3. LVADs

Left ventricular assist devices (LVADs) remain a pivotal therapeutic strategy for end-stage heart failure (HF). According to the European Society of Cardiology (ESC)’s latest HF guidelines, long-term mechanical circulatory support with an LVAD is indicated in patients with advanced HF with reduced ejection fraction, with the persistence of severe symptoms despite optimal medical and device therapy, and without severe right ventricular dysfunction and/or severe tricuspid regurgitation. This could be either a destination therapy in case the patient is not eligible for heart transplantation or a “bridge” to help the patient reach the procedure if he is waiting for a transplant [64].

LVADs have been considerably evolved over the last years, but their main function is to actively pump blood from the failing left ventricle and through an electrical motor to return it to the ascending aorta, actually bypassing the left ventricle and taking on its workload, thus unloading the “wounded” myocardium [65]. We are already in the third generation of LVADs, now offering continuous blood flow, reduced size, and fewer complications compared to the older devices. The main representatives of the currently used devices are the HeartMate II (Thoratec, Pleasanton, CA, USA; St. Jude, Memphis, TN, USA; Abbott, Abbott Park, IL, USA), the HeartWare HVAD (HeartWare, Medtronic, Minneapolis, MN, USA), and the HeartMate 3 (St. Jude, Memphis, TN, USA; Abbott, Abbott Park, IL, USA) [65].

The implantation of an LVAD can lead to an enhanced cardiac output and index, followed by reduced adrenergic stimulation, better tissue perfusion, and a boost in physical activity [58]. Post LVAD, there is a proven increase in blood flow reaching the pancreas, resulting in better glucose homeostasis and reduced insulin resistance in both diabetic and non-diabetic patients [7]. Moreover, research demonstrated that LVADs can immensely improve HbA1c levels, reduce daily insulin requirements in diabetic patients, and decline fasting blood glucose levels, without affecting BMI, in both diabetic and non-diabetic patients [7,8]. Daily insulin doses are shown to be closely related to age. In other words, younger patients are prone to a bigger decrease in average daily insulin requirements in comparison to older patients. Bearing in mind that HF is associated with insulin resistance, it is likely that the implantation of an LVAD also prevents patients with HF from potential DM development [8].

There are studies indicating its significant role in diabetic cardiomyopathy and its impact on the molecular pathways. The speculated pathophysiology includes endocrine and paracrine abnormalities, inflammation markers and oxidative stress, and disrupted intramuscular Ca^2+^ handling. It is a fact that GH resistance accompanied by low levels of IGF-1 develops in chronic inflammatory states and is correlated with catabolic syndromes, such as advanced HF. The endocrine pathway strongly associated with HF is the GH/IGF-1 signaling one, which can deteriorate skeletal muscle function and the regulation of lipolysis and lipid oxidation. More specifically, HF is characterized by elevated GH levels, which indicate GH resistance, and lowered IGF-1 levels, as well as IGFBP-3 levels (insulin-like growth factor binding protein-3). Higher GH levels indicate an elevated GH/IGF-1 ratio (the marker for GH resistance) as well. Moreover, GH resistance has been associated with inflammation tumor necrosis factor-alpha and interleukin-6-mediated inhibition of hepatic GH signaling as well as levels of angiotensin II [66].

Research has shown that IGF-1 levels and muscle mass in HF are expected to increase through GH supplementation and aerobic exercise training; yet, still, GH resistance can only be faced with cardiac transplantation. The implantation of an LVAD can also assist this pathway. Research has proven that LVAD placement in patients with HF leads indeed to lower levels of circulating GH, but the levels of IGF-1 and IGFBP-3 were not affected and remained suppressed. Therefore, the GH/IGF-1 ratio diminished following LVAD implantation. We ought to mention that even though the levels improved, they were still not normal in comparison to controls. In other words, in HF, the levels of GH are elevated in comparison to controls, and they tend to remain this way, even after LVAD placement.

Moreover, the placement of an LVAD assists in improving skeletal muscle function. There is a post-LVAD increase in muscle fiber cross-sectional area (CSA) and improved skeletal muscle oxidative function [66]. After a fatty acid oxidation analysis of skeletal muscle tissue from patients, both before and after LVAD implantation, it was evident that there was an increase in the production of oleic acid post LVAD [66], which was attended by elevated phosphorylation of Akt in the skeletal muscle of patients.

Other than the above, the LVAD resulted in higher expression in muscles of the insulin-independent glucose transporter type 4 (GLUT4) and, therefore, greater glucose transport capacity [66]. Other changes include a decrease in pyruvate dehydrogenase kinase-4 (PDK-4) by 50% and an increase in cluster of differentiation (CD) 36 in carnitine palmitoyl transferase (CPT)-1, which is a mitochondrial fatty acid uptake transporter, and in the levels of peroxisome proliferator-activated receptor γ co-activator 1α in the rectus abdominus muscle of patients (PGC1a).

It is important to mention that the implantation of an LVAD is a surgical procedure that causes major stress to the body and could trigger multiple responses [8]. Therefore, it is crucial that the clinicians dealing with these patients apply a meticulous follow-up plan post-procedurally. It is also vital that possible decreased insulin requirements post LVAD is taken into consideration and insulin administration adjusted accordingly.

## 11. Conclusions

Heart failure is an early and severe complication of diabetes mellitus. The endocrine system and the heart are two interrelated entities, as this is proven by the close relationship between insulin and cardiac function. Insulin significantly contributes to cardioprotection via multiple pathways and various subsequent downstream proteins. Even slight malfunction in the participating pathways can lead to myocardial dysfunction, resulting finally in overt heart failure. At this stage, the implantation of an LVAD and its contribution to the regulation of the neuromodulatory effects of insulin on the heart is pivotal and may decelerate, stabilize, or even revert the deleterious cascades that have been activated in end-stage heart failure.

## Data Availability

Not applicable.
